# Reactive Oxygen Species Drive Epigenetic Changes in Radiation-Induced Fibrosis

**DOI:** 10.1155/2019/4278658

**Published:** 2019-02-06

**Authors:** Shashank Shrishrimal, Elizabeth A. Kosmacek, Rebecca E. Oberley-Deegan

**Affiliations:** Department of Biochemistry and Molecular Biology, University of Nebraska Medical Center, Omaha, NE 68198, USA

## Abstract

Radiation-induced fibrosis (RIF) develops months to years after initial radiation exposure. RIF occurs when normal fibroblasts differentiate into myofibroblasts and lay down aberrant amounts of extracellular matrix proteins. One of the main drivers for developing RIF is reactive oxygen species (ROS) generated immediately after radiation exposure. Generation of ROS is known to induce epigenetic changes and cause differentiation of fibroblasts to myofibroblasts. Several antioxidant compounds have been shown to prevent radiation-induced epigenetic changes and the development of RIF. Therefore, reviewing the ROS-linked epigenetic changes in irradiated fibroblast cells is essential to understand the development and prevention of RIF.

## 1. Introduction

Fibrosis is characterized by an aberrant accumulation of extracellular matrix (ECM) proteins that result in the loss of normal tissue and organ function [1]. It is a significant cause of morbidity and mortality worldwide [2–9]. Exposure to radiation can trigger a condition known as radiation-induced fibrosis (RIF). The cell type involved in developing fibrosis is the myofibroblast, which primarily arises from fibroblasts upon radiation. Myofibroblasts can also arise from other cell types through the process of differentiation or by epithelial/endothelial-mesenchymal transitions [1]. Under normal conditions, myofibroblasts play a critical role in normal wound closure after injury [10]. After wound healing and restoration of ECM to homeostatic levels, the myofibroblasts undergo apoptosis [1]. However, wounds that fail to heal correctly contain persistent myofibroblasts that leave a keloidal or hypertrophic scar. These active myofibroblast cells do not undergo apoptosis after healing and continue to damage the tissues and organs by producing excessive amounts of ECM proteins. The persistent nature of an activated myofibroblast is maintained through molecular feedforward loops by autocrine and paracrine signaling and the influx of inflammatory cells [11, 12]. Reactive oxygen species (ROS) are one such signal that helps maintain the myofibroblast phenotype [13].

Ionizing radiation used in cancer therapy includes high-energy gamma rays and X-rays, which have sufficient energy to displace electrons from atoms. Interaction of these waves with water molecules leads to the excitation and ionization of water to form free radicals and ROS that include e_aq_^−^, hydroxyl radicals (^•^OH), hydroperoxy radicals (HOO^•^), hydrogen peroxide (H_2_O_2_), and superoxide (O_2_^•−^) [13]. Generation of ROS also leads to an acute increase in oxidative stress within cells following radiation [14]. ROS can increase the levels and activity of several prooxidant enzymes, such as NADPH oxidases (NOXs), cyclooxygenases (COXs), nitric oxide synthases (NOSs), and lipoxygenases (LOXs) [15], which further promote ROS generation and the development of RIF. In addition to ROS, reactive nitrogen species (RNS), such as peroxynitrite (ONOO^−^), are also generated and result in changes to signaling pathways, gene transcription, mitochondrial functioning, metabolism, and the chromatin architecture.

RIF is often observed in patients that have undergone radiation therapy for cancer treatment and persists long after the initial exposure to radiation [16]. RIF reduces the quality of life of patients after treatment [2–8], and there are no safe, approved therapies to mitigate this problem. Hence, the focus on understanding the ROS-mediated changes in chromatin-modifying proteins that lead to the development of RIF is essential. We will review the differences in expression and posttranslational modifications of chromatin regulators caused by ROS generated after radiation exposure. These changes could serve as biomarkers to estimate the severity and susceptibility of patients to develop RIF after radiation therapy. In some cases, epigenetic regulation has not been studied in the context of RIF. Therefore, we will review the reported changes in other fibrotic conditions. Lastly, we will discuss the potential of antioxidant drugs and epigenetic inhibitors used to prevent the development of RIF.

## 2. ROS-Mediated Metabolic Changes in RIF

The mitochondria are essential cell organelle involved in regulating both metabolism and ROS levels that impact the epigenome. Under normal metabolic conditions, the mitochondria produce low basal levels of superoxide via the electron transport chain, which is required for normal cellular signaling. Through normal metabolism, the mitochondria can also regulate the generation of epigenetic metabolites such as nicotinamide adenine dinucleotide (NAD), *α*-ketoglutarate (*α*-KG), S-adenosyl methionine (SAM), and acetyl-CoA. These molecules serve as cofactors for several epigenetic proteins and control epigenetic modifications such as DNA or histone methylation, histone acetylation, and ADP-ribosylation. Therefore, damage to the mitochondria can increase both levels of ROS and epigenetic metabolites, thereby promoting epigenetic alterations in the nucleus.

**Figure 1:**
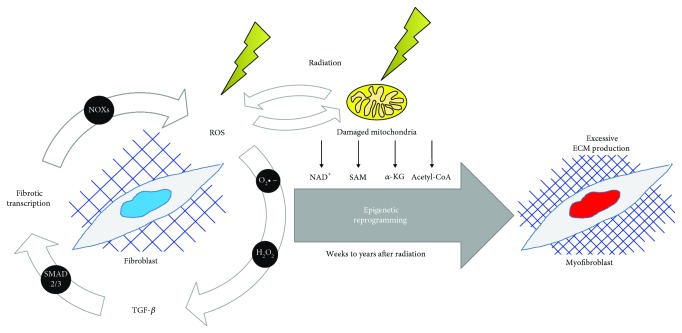
Radiation induces reactive oxygen species (ROS) generation, which drives epigenetic changes in fibroblast cells. ROS can be directly generated due to radiation exposure and through the damage of mitochondria. This leads to the activation of the TGF-*β* signaling pathway, which sustains an increase in ROS levels by increasing NOX4 expression, thereby setting up a vicious cycle of high oxidative stress, which drives epigenetic reprogramming of fibroblast cells to myofibroblasts. Further, damaged mitochondria have altered production of redox-sensitive epigenetic metabolites that serve as cofactors for chromatin-modifying proteins. NOXs: NADPH oxidases; NAD^+^: nicotinamide adenine dinucleotide; SAM: S-adenosylmethionine; *α*-KG: *α*-ketoglutarate; ECM: extracellular matrix.

Ionizing radiation can directly damage mitochondrial DNA and nuclear DNA that codes for mitochondrial proteins, which leads to several functional changes in the mitochondrial structure, activity, and function [17–19]. Radiation exposure can result in excessive production of mitochondrial ROS due to an increase in mitochondrial abundance and loss in mitochondrial membrane integrity/potential [17, 20, 21]. Further, radiation-induced mitochondrial damage reduces production of the tricarboxylic acid (TCA) metabolites and causes a slight increase in fatty acid metabolism. Alteration of global metabolism and changes in the production of epigenetic metabolites or cofactors for chromatin-modifying proteins results in the modification of the fibroblast epigenome [22]. Also, antioxidant molecules, such as glutathione and NAD^+^, are significantly reduced following radiation and remain reduced for many hours following radiation exposure. As reported, many of the depleted metabolites are associated with oxidative stress and DNA repair pathways [23]. Thus, epigenetic changes in fibroblast cells and the development of RIF can be influenced by the changes in ROS and metabolism affected by damaged mitochondria as shown in Figure 1.

## 3. ROS-Mediated TGF-*β* Signaling Changes in RIF

The impact of ROS on TGF-*β* signaling is the most studied in the context of RIF [24–27]. An increase in ROS after radiation exposure leads to the activation of the TGF-*β* signaling pathway through the oxidation of cysteine residues of the latency-associated peptide (LAP). Oxidation of LAP leads to a conformational change in LAP, which allows the release of TGF-*β* from the latent complex. An active TGF-*β*, upon binding to TGF-*β* receptors, leads to the phosphorylation and activation of transcription factors, such as Smad2 and Smad3 [28]. As shown in Figure 1, it is known that ROS and TGF-*β* are interlinked by both feedforward and feedback mechanisms [25, 29]. TGF-*β* stimulation increases the basal level of ROS through several NADPH oxidases (NOXs), including NOX4, via the canonical Smad2/3 signaling factors [30] and activation of PI3K [28, 31]. Generation of ROS through NOX4 upregulation can also lead to the activation of the noncanonical Smad signaling pathway, which includes the activation of c-Src and FAK kinases [32]. These changes in the TGF-*β* signaling pathway can also crosstalk with the PI3K/AKT signaling pathway that leads to changes in the epigenome and the development of fibrosis.

## 4. ROS-Mediated DNA Methylation Changes in RIF

The covalent addition of methyl (CH_3_) groups to DNA is controlled by DNA methyltransferases (DNMTs). In general, an increase in DNA methylation or hypermethylation of CpG islands at gene promoters is responsible for suppression of gene transcription. DNMTs can transfer methyl groups from SAM, and other methyl donors, to cytosines in DNA. The three enzymes involved in DNA methylation are DNMT1, DNMT3a, and DNMT3b. DNMT1 is a maintenance enzyme that copies methylation patterns onto an existing or new DNA strand following replication. DNMT3a and DNMT3b are classified as de novo DNMTs and are not dependent on preexisting methylation marks on DNA strands.

Aberrant DNA methylation is responsible for myofibroblast activation and changes in expression of fibrotic genes [32–34]. Changes in expression of DNMT1 [35, 36], DNMT3a [36, 37], and DNMT3b [36] have been identified in different models of fibrosis [38–40]. Upregulation of DNMT1 can be detected in fibrotic skin, kidneys, lungs, and liver tissues [32, 35, 41, 42]. Both DNMT1 and DNMT3a protein expression were found to be upregulated following 15 Gy irradiation of lung fibroblast cells [35]. This *in vivo* upregulation of DNMT1 and DNMT3a was observed at six weeks postradiation and was maintained up to six months following radiation exposure [35]. In contrast, fractionated low-dose radiation exposure leads to a small decrease in DNMT1 and DNMT3a expression, along with a reduction in methyl-CpG-binding protein MeCP2 [43]. This change in DNMT levels causes hypermethylation of antifibrotic genes: RASAL1 [44–50], PTCH1 [34, 51] PPAR-*γ* [52], SOCS1/3 [53, 54], DKK1 [55], E-cadherin [56], p14 (ARF) [57, 58], FlI1 [59], Thy-1 [40], PTGER2 [60], and hypomethylation of profibrotic gene promoters: TGF-*β*1 [44], Smad4/7 [61–63], TP53 [64–66], MMP7 [67], and SPP1 [68]. Therefore, the expression of DNMT with radiation exposure is dependent on the cell type, radiation dose, tissue type, and sex of the organism as reported by Raiche et al. [69].

Changes in the levels of DNA methyltransferases are closely associated with the TGF-*β* signaling pathway [32, 40, 44, 70]. Alternatively, crosstalk of the TGF-*β* signaling pathway with the PI3K/Akt pathway can also increase DNMT expression via a transcription-independent mechanism involving an increase in phosphorylation and inactivation of glycogen synthase kinase-3*β*, leading to a decrease in ubiquitination of DNMT1 [32]. Increase in DNMT3a is attributed to an increase in protein translation due to the activation of the mammalian target of rapamycin complex 1 by Akt [32]. This reported mechanism has been studied in the context of activation and differentiation of fibroblast cells but not in the context of radiation exposure.

Inhibition of DNMTs using 5-aza-2′-deoxycytidine [37, 64, 71] or siRNA-mediated knockdown of DNMT1 expression prevents the activation of fibroblast cells and hepatic stellate cells [16, 37] and protects against the development of fibrosis. This reduction in activated fibroblast cells is also associated with a reduction in ROS levels [72–74]. Moreover, the addition of hydrogen peroxide to embryonic lung fibroblasts rapidly increases DNMT levels [35]. Conversely, decreasing oxidative stress, using a superoxide scavenger Mn (III) TBAP [35], N-acetylcysteine [75], or L-NAME (NOS inhibitor) [75], resulted in decreased DNMT1 levels and loss of global DNA methylation. Therefore, it is suspected that superoxide and hydrogen peroxide are the ROS intermediates involved in the regulation of DNMT in RIF.

In certain cell types, such as cardiac fibroblast cells, stimulation with recombinant TGF-*β* leads to downregulation of DNMT1 and DNMT3a expression and inhibition in global DNMT activity [76]. This has been linked to a decrease in DNA methylation at the promoter of COL1A1 and an increase in the expression of COL1A1 mRNA [76]. Therefore, changes in expression of DNMT proteins and changes in DNA methylation by the direct activation of the TGF-*β* signaling pathway or indirect activation through radiation and ROS can be variable and dependent on the tissue and organ under investigation.

Along with an increase in levels of DNMTs, an increase in the methylated DNA-binding protein, MeCP2, is also observed during fibrosis [77, 78]. Binding of MeCP2 to methylated CpG regions causes transcriptional repression. Similar to DNMT1, expression levels of MeCP2 are sensitive to changes in oxidative stress and redox balance [79–82]. It is believed that MeCP2 levels increase to maintain DNA methylation by the formation of DNMT1-MeCP2 complexes in an increasingly oxidative environment of fibrosis [83, 84]. Fractionated low-dose radiation exposure has been reported to cause an increase in MeCP2 in the brain [85] and downregulation in the spleen [86] and thymus [43]. Upregulation of MeCP2 was found to be associated with downregulation of antifibrotic genes, such as PPAR-*γ* [87], RASAL1 [88], and PTCH1 [34, 88], thereby promoting myofibroblast differentiation and the development of fibrosis [87].

Some of the DNA methylation changes at specific gene promoters may be independent of changes in the expression of DNMTs. This is because it is suggested that superoxide is a strong anion that can participate in nucleophilic substitutions and free radical abstraction, leading to changes in DNA methylation and histone modifications. Superoxide neutralizes positive charges of methyl donors, SAM, and acetyl-CoA, which can then deprotonate the cytosine molecule at the C-5 position and accelerate the reaction of DNA with SAM; thereby, causing methylation of DNA [89, 90]. However, this has not been tested in the context of fibrosis.

In summary, increased oxidative stress after radiation is intimately interconnected with increased DNMT levels, activity, and DNA methylation. Activation of the TGF-*β* signaling pathway by ROS mechanistically drives the sustained high levels of DNMTs. Further, changes in interaction with binding partners (MeCP2, HMTs, and HDACs) and cofactors (SAM) can lead to changes in DNMT levels and DNA methylation at specific gene promoters. Targeting DNMTs, the TGF-*β* signaling pathway, or oxidative stress has been shown to modulate DNA methylation and reduce fibrosis. However, large-scale genome-wide DNA methylation studies are needed to delineate hypomethylation and hypermethylation status at different gene promoters during RIF.

## 5. ROS-Mediated Histone Modification Changes in RIF

Histones can be modified through covalent posttranslational modifications (PTMs) that control the open or closed architecture of the chromatin for gene expression. These modifications include methylation, acetylation, phosphorylation, ubiquitylation, and sumoylation. Changes in histone modifications have been associated with altered expression of profibrotic and antifibrotic genes that lead to fibrosis. Furthermore, changes in the expression of microRNAs have also been associated with histone modifications and fibrotic gene expression. PTMs such as histone acetylation and histone methylation marks are redox sensitive and are inherited by daughter cells in RIF.

### 5.1. Role of Histone Acetylation in RIF

Histone acetylation is regulated by histone acetyltransferases (HATs) and histone deacetylases (HDACs). The balance between the epigenetic marks added by HATs and removed by HDACs helps to control gene transcription. In general, acetylated histones are associated with transcriptionally active chromatin and deacetylated histones with inactive chromatin [87].

HATs are enzymes that catalyze the transfer of an acetyl group from acetyl-CoA to the *ε*-amino group of histone lysine residues. Out of the 30 known HAT enzymes, only EP300 (p300) and CREBBP (CBP) have been reported to play a role in RIF [91]. Levels of p300/CBP were found to be significantly elevated in skin fibroblast cells 12 hours after radiation exposure but not after 24 or 36 hours [91]. This increase in p300/CBP also correlated with an increase in alpha-smooth muscle actin (*α*SMA), which is a marker for myofibroblast cells.

The mechanism of p300/CBP upregulation and/or increased activity is also linked to an active TGF-*β*/ROS signaling pathway [91–100]. p300 is a direct transcriptional target of TGF-*β* signaling and is known to form a feedforward loop with an active TGF-*β* signaling pathway [101–103]. Interaction of p300 with Smad3 is essential for the TGF-*β*-mediated synthesis of collagen [101]. Also, inhibition of p300 expression or activity reduces fibrosis [96, 100, 104–107]. The role of p300 in fibroblast biology and fibrosis has been studied by Ghosh et al., and the targeted disruption of p300-mediated histone acetylation has been proposed as a viable antifibrotic strategy [101].

The redox environment can directly alter the activity of p300 due to the oxidation of key cysteine residues. Specifically, the oxidation of these thiols results in reduced p300 activity. Redox-active compounds such as MnTE-2-PyP and hydroxynaphthoquinones can downregulate p300 activity [108–111]. The use of alpha-lipoic acid, a dietary antioxidant supplement, has been shown to protect against RIF in mice by downregulating expression and activity of p300/CBP [112–115]. Similarly, inhibition of p300 activity using curcumin also reduces cardiac fibrosis and hypertrophy [32, 94, 116]. However, thiol oxidation of p300 during RIF has not been studied.

Both p300 and CBP have high sequence homology and can act as transcriptional coactivators, which recruit basal transcriptional machinery, including RNA polymerase II, to gene promoters. p300 and CBP promote the transcription of fibrotic genes, such as matrix metalloproteinase-2 (MMP2), matrix metalloproteinase-9 (MMP9), *α*SMA, and plasminogen activator inhibitor-1 (PAI-1) [91] in this manner. Moreover, increased histone acetylation at the H3K9/14 and H3K18 marks has been associated with an upregulation of TGF-*β*1, TGF-*β*3, and another potent profibrotic factor, connective tissue growth factor (CTGF) [117].

During fibrosis, an increase in histone acetylation can also be mediated by an increase in activity of ATP citrate lyase (ACL), an enzyme that converts citrate to acetyl-CoA, which is a substrate for HATs [117]. Thus, histone acetylation is affected by changes in glucose metabolism and oxidative stress during fibrosis [118, 119]. Correspondingly, high-glucose treatment can increase oxidative stress and increase pan-H3 histone acetylation marks [108, 120]. However, this process has not been studied in the context of RIF.

In summary, histone acetylation in RIF is attributed to an increase in the level of expression and activity of HAT enzymes, p300 and CBP. HAT expression is further upregulated by the TGF-*β* signaling pathway. Antioxidants have been shown to inhibit HAT activity and prevent the development of fibrosis. However, the mechanism of inhibition of HAT activity by antioxidants has not been determined in the context of RIF. Other studies, unrelated to fibrosis, point towards susceptibility of p300 to several PTMs that are influenced by a change in the oxidative environment [101, 108, 121, 122].

### 5.2. Role of Histone Deacetylation in RIF

HDACs are a class of enzymes that compress the chromatin by removing acetyl groups, which results in a downregulation in gene expression. There are a total of 11 known HDACs that are dependent on the cofactor, Zn^2+^, to deacetylate histones. Another class of enzymes known as sirtuins (Sirt) contains seven members that deacetylate histones and are dependent on NAD^+^ as a cofactor.

Upregulation of several HDAC enzymes is known to be in involved in the development of fibrosis [123–131]. Profibrotic stimulation, using TGF-*β* or the platelet-derived growth factor (PDGF), upregulates the expression of HDAC1, HDAC2, and HDAC4, which results in fibrosis of a variety of tissues [124, 125, 132]. Also, all three HDAC proteins involved in fibrosis are redox sensitive. Upregulation of certain HDACs can lead to the deacetylation of histones associated with antifibrotic genes and downregulation of genes that prevent the development of fibrosis. Hence, HDAC proteins are reported to be potential targets for fibrotic disorders [133]. However, the role of HDAC proteins and HDAC inhibitors in RIF has not been studied.

HDAC1, a well-known epigenetic and cell cycle regulator, is redox sensitive and plays a crucial role in normal development and tumor progression [134, 135]. During fibrosis, HDAC1 upregulation causes epithelial-mesenchymal transition by suppressing the transcription of ZO-1 and E-cadherin [124]. In addition, HDAC1 promotes fibrosis by inhibiting the expression of the antifibrotic Smad7 protein in renal fibrosis [95]. In agreement with this finding, the HDAC inhibitor, suberoylanilide hydroxamic acid, was successful in stabilizing Smad7 levels, thereby preventing fibroblast differentiation and collagen expression in a lung fibrosis model in rats [123].

Similarly, HDAC4 upregulation enhances the expression of profibrotic genes in lung fibrosis [136, 137] and causes transdifferentiation of hepatic stellate cells to myofibroblast cells [138]. Knockdown of HDAC4 inhibits fibrosis by reversing the TGF-*β*-stimulated transformation of fibroblasts to myofibroblasts [139]. HDAC4 is a redox-sensitive protein, where oxidation of Cys^667^ and Cys^669^ affects its activity and is independent of other phosphorylation modifications [140, 141]. Specifically, reduction of these two cysteine residues has also been shown to prevent its nuclear export [141].

In liver fibrosis, HDAC2 was found to be upregulated, which activates hepatic stellate cells through the suppression of the antifibrotic protein, Smad7 [142]. Moreover, HDAC2 and DNMT1 have been suggested to cooperate in adding repressive chromatin marks at gene promoters to suppress the expression of antifibrotic genes, such as RASAL1 [46, 143]. Oxidative stress causes tyrosine nitration of HDAC2, thereby reducing its activity [144]. These PTMs are prevented with the use of antioxidants, such as glutathione monoethyl ester or polyphenol-curcumin [145, 146]. Overexpression of SOD2 decreases HDAC2 expression due to an increase in ubiquitination of HDAC2 molecules [147]. Therefore, a change in expression and activity of HDAC2 is highly regulated by the redox environment [148–151].

Reduction in HDAC1/2 expression using gallic acid or valproic acid sodium (VPA) attenuates hypertension, cardiac remodeling, and fibrosis in mice [152]. RNS, such as nitric oxide, has an inhibitory effect on HDAC activity resulting in the hyperacetylation of specific genes [153]. The inhibitory effects of RNS on HDAC proteins are associated with nitrosylation of tyrosine residues and aldehyde-adduct formation on HDAC1, HDAC2, and HDAC3 proteins [145]. As mentioned previously, PTMs of HDACs due to oxidative modification of conserved cysteine residues have also been linked to nuclear export [154]. However, these changes mediated by RNS have not been studied extensively in the context of RIF.

HDAC inhibitor (HDACi) drugs, romidepsin [155], trichostatin A [156, 157], suberoylanilide hydroxamic acid [123, 158], sodium valproate [159], panobinostat [160, 161], and valproic acid [162, 163], have all been shown to suppress fibrosis. In a standard animal model of cutaneous radiation syndrome, application of topical formulations of phenylbutyrate, an HDACi [164] and oxidative stress inhibitor [165–167], reduced acute skin damage and protected from late radiation-induced effects, such as fibrosis and tumor formation [168]. This reduction in RIF after HDAC inhibition further correlated with suppression of TGF-*β* and TNF-*α* signaling [168]. Therefore, HDAC inhibitors have been used and are proposed as radioprotectors for treating RIF [168]. However, the potential nonspecificity of these broad inhibitors may produce many unwanted side effects, making these drugs potentially unsuitable for therapeutic use.

In summary, HDACs are upregulated during radiation and are associated with fibrosis but vary with the tissue type and radiation dose. The majority of upregulated HDAC proteins during fibrosis can be countered with the use of either HDACi or antioxidants. Some changes in PTMs of HDAC proteins due to oxidative stress have been associated with changes in HDAC activity but have not been studied in the context of RIF.

### 5.3. Role of Sirtuin Deacetylases in RIF

Sirtuin proteins are deacetylase enzymes that are redox sensitive because they require NAD^+^ as a cofactor to be active. As mentioned above, radiation-associated damage to the mitochondria can alter levels of NAD^+^, which can change the activity of sirtuin proteins. These enzymes are involved in the deacetylation of both histone and nonhistone proteins depending on their localization. Sirt1, Sirt6, and Sirt7 localize to and exert distinct deacetylation functions in the nucleus [169], while Sirt3, Sirt4, and Sirt5 localize to the mitochondria [170] and are indirectly involved in epigenetic reprogramming during fibrosis and are involved in the modulation of oxidative stress by regulating mitochondrial antioxidant proteins and cellular metabolism.

In contrast to HDACs, Sirt1 overexpression or upregulation protects against fibrosis by attenuating the TGF-*β* and NF-*κ*B signaling pathways [32, 92, 171–180]. Moreover, Sirt1 is a negative regulator of p300 expression [92, 181]. Ionizing radiation, cigarette smoke extract, and carbon tetrachloride increase oxidative stress and downregulate Sirt1 gene expression [32, 182, 183]. Nonionizing radiation, such as UV irradiation, also decreases Sirt1 activity [184], which may result in fibrosis. This change in Sirt1 activity needs to be further investigated in relation to the cellular NAD^+^ levels [184] and oxidative stress-dependent NAD^+^ metabolism [185] during fibrosis. The decrease in Sirt1 expression, activity, and changes in its subcellular localization can be linked to changes in Sirt1-catalyzed PTMs influenced by oxidative stress [32, 148, 186–189]. Treatment of fibroblast cells with H_2_O_2_ downregulates Sirt1 levels [190], while the use of antioxidants such as resveratrol [191–197], curcumin [198], phenylephrine [182], and vitamin D [199, 200] has been shown to upregulate Sirt1 expression after radiation.

To combat and repair the cell from radiation-induced oxidative damage, fibroblast cells upregulate and/or increase the activity of Sirt1 [171, 172, 192, 201–203]. Sirt1 knockdown and overexpression have been shown to alter ROS levels within a variety of cell types [203–207]. Sirt1 is involved in deacetylation of histones, specifically the removal of H3K9Ac, H3K14Ac, H4K16Ac, and H1K26Ac marks, which leads to an upregulation of antioxidant genes such as superoxide dismutase (SOD) [148]. Further, deacetylation of transcriptional factors such as the nuclear factor erythroid-related factor (Nrf), Nrf1 or Nrf2 [208, 209], and peroxisome proliferator-activated receptor gamma coactivator-1 alpha (PGC1-*α*) [174, 176, 178, 210–212] is also involved in controlling the expression of SOD. The Nrf2 transcription factor is a crucial regulator of the antioxidant defense pathway and has been reported to inhibit the TGF-*β* signaling pathway [208]. Therefore, Sirt1 is a redox sensor that acts as an antifibrotic protein via deacetylation of both histones and nonhistone proteins.

Similarly, Sirt3, Sirt6, and Sirt7 are all redox-sensitive proteins and modulate oxidative stress in fibrotic tissues. Sirt3 upregulation has a protective effect against radiation-induced lung injury by exerting anti-inflammatory and antioxidative properties [213–215]. Further, Sirt3 is responsible for preventing epithelial-mesenchymal transition (EMT) by elevating the levels of Nrf2 and PGC1-*α* expression [216, 217]. In parallel to this, Sirt3 deficiency has been shown to promote lung fibrosis [214] and its activity is required to deacetylate and activate MnSOD. An active MnSOD enzyme is necessary to detoxify mitochondrial ROS and prevent mtDNA damage [214]. Sirt6 overexpression prevents hepatic fibrosis by curbing inflammation and oxidative stress [218], and Sirt6 deficiency results in progressive renal inflammation and fibrosis [219]. Moreover, it is known that Sirt6 exhibits an inhibitory effect on the activity of TGF-*β* [220] and NF-*κ*B signaling [221] that are activated in RIF. In addition, a decrease in expression of Sirt7 is associated with the development of lung fibrosis [222, 223] and fibroblast differentiation in cardiac tissue [216].

Paradoxically, Sirt2 and Sirt4 downregulation prevents fibrosis and is also modulated by treatment of antioxidant molecules [224, 225]. Sirt2 potentiates radiation-induced damage in fibroblast cells by interacting with *β*-catenin and, thereby, inhibiting Wnt signaling [226]. Inhibiting Sirt2 activity prevents transformation and preserves the integrity of aging fibroblast cells against ROS [226]. However, in the brain, Sirt2 has been shown to be essential in preventing neurotoxicity and cognitive dysfunction after whole brain radiation [227] and plays a role in preventing neuroinflammation and brain injury [228]. Sirt4 is involved in the development of cardiac fibrosis after angiotensin II treatment and is involved in the regulation of oxidative stress [229]. Treatment with a SOD mimetic, 5, 10, 15, and 20-tetrakis-(4-benzoic acid) porphyrin, inhibited ROS accumulation and Sirt4-mediated development of cardiac fibrosis [229].

In contrast to the upregulation of HDAC proteins, upregulation of most sirtuins protects from RIF development. The upregulation and increase in activity of sirtuins combat radiation-induced oxidative stress and counterbalance the increase in expression of HAT enzymes and radiation-induced epigenetic modifications. Like HATs, increase in sirtuin protein levels or activity occurs through acute changes in signaling pathways, redox environment, and metabolite production after radiation. The protective effects of sirtuin proteins are thought to be mediated, in part, by deacetylating histones and key transcription factors involved in the antioxidant pathway, such as Nrf2 and PGC1-*α*.

### 5.4. Role of Histone Methylation in RIF

Histone methylation can either increase or decrease transcription of genes depending on the amino acid methylated (lysine or arginine), position on the histone tail, and the number of methyl groups added. This dynamic process is regulated by more than 40 histone methyltransferases (HMTs) and demethylases, which are involved in the establishment of a histone methylome. For these reasons, specific histone methylation alterations have not been studied in the context of radiation. However, reports indicate that histone methylation plays a critical role in fibrotic gene expression and fibrosis [230].

TGF-*β* stimulation increases the expression of EZH2, SET7 [231], SET9 [231], and G9a [232]. Furthermore, an active TGF-*β* pathway has been linked to an increase in H3K4Me1, H3K4Me2, and H3K4Me3 (active chromatin marks) and a decrease in H3K9Me2 and H3K9Me3 (repressive chromatin marks) at profibrotic gene promoters [230, 231, 233, 234]. Among the several HMTs, EZH2 was shown to be upregulated during the differentiation of fibroblasts to myofibroblasts in the lungs of patients with idiopathic pulmonary fibrosis [235]. Induction of EZH2 expression after TGF-*β* stimulation can lead to an increase in H3K27Me3 (repressive marks) at COX-2 gene promoters (antifibrotic gene), which promotes fibrosis [236, 237]. This increase in EZH2 expression also correlates with an increase in the expression of ECM proteins, such as COL3A1 [233]. Importantly, antifibrotic genes, such as Caveolin-1 [238], are exclusively regulated by histone methylation [239] and not by DNA methylation. Further, EZH2 forms repression complexes with MeCP2 and SIN3A, transcriptional repressors, which can suppress the expression of antifibrotic genes [87, 240]. Treatment of epithelial cells with H_2_O_2_ causes the translocation of EZH2 from the nucleus to the cytoplasm by regulating its phosphorylation status [241]. Inhibition of HMTs, using 3-deazaneplanocin A (DZNep), suppressed the progression of renal and pulmonary fibrosis [242, 243]. Further, inhibition of the TGF-*β* and TNF-*α* signaling pathways using a novel indole compound, MA-35, resulted in the attenuation of renal inflammation and fibrosis by decreasing H3K4me1 histone modification at the COL1A1 and PAI-1 fibrotic gene promoters [244]. Inhibition of H3K9me1 using BIX01294, an inhibitor of G9a methyltransferase, prevented the development of renal fibrosis by maintaining expression of the antifibrotic gene, Klotho [232]. Therefore, an active TGF-*β* pathway, due to the generation of ROS after radiation, can lead to an upregulation of these HMTs leading to the development of RIF [232].

Activation of hepatic stellate cells (HSC), by bile duct ligation procedure, leads to transdifferentiation of HSC to a myofibroblast-like phenotype [245]. This transdifferentiation is associated with an increase in HMTs such as KMT2H (aka ASH1), KMT1A (aka SUV39H1), KMT1B (aka SUV39H2), KMT1D (aka GLP), KMT6 (aka EZH2), KMT3C (aka Smyd2), KMT2A (aka MLL1), KMT2E (aka MLL5), and KMT2F (aka SET1A) and a compensatory increase in histone demethylases (HDMs) such as KDM1 (aka LSD1), KDM5B (aka JARID1b), KDM4A (aka JMJD2a), and KDM4B (aka JMJD2b) [245]. This is also associated with the upregulation of profibrotic genes, such as *α*SMA, TIMP-1, collagen I, and TGF-*β*. Several of these methylase enzymes are activated and inhibited by metabolic cofactors that are considered redox intermediates such as NAD+, SAM, flavin adenine dinucleotide (FAD), and 2-oxoglutarate. Further, the jumonji domain-containing (jmjC) family of proteins, which is involved in histone demethylation, is highly redox sensitive due to the presence of a transition metal, iron (Fe), at the enzyme active site. Fe (II) is used as a cofactor for the histone demethylation reaction and can interact with H_2_O_2_ to produce ·OH, leading to an increase in oxidative damage and histone methylation [246, 247]. Changes in the redox environment have also been reported to increase the activity of LSD1, which is involved in DNA repair after oxidative damage [248]. HDMs, such as KDM6B, can be induced by the TGF-*β* pathway and promote EMT transition during fibrosis [249], which can also have implications in the context of RIF. However, the role of these histone methylation-regulating proteins has not been extensively studied in the context of changing oxidative stress and fibrosis.

## 6. ROS-Mediated Noncoding RNA Changes in RIF

Noncoding RNAs that regulate epigenetic processes in RIF include, micro-RNAs (miRs), long noncoding RNA (lncRNA), and circular RNA (circRNA). miRs are considered to play an essential role in regulating the epigenome and are modulated by changes in oxidative stress during radiation exposure [26, 250]. Further, expression of miRs is interconnected with the TGF-*β* signaling pathway [16, 36, 132, 250–259]. DROSHA and DICER regulate the biogenesis of the majority of miRs in healthy cells and are involved in radiation damage responses due, in part, to the production of ROS [260]. Increase in ROS inactivates DROSHA and DICER, which impairs DNA damage responses in human fibroblasts after radiation [261]. TGF-*β* signaling pathway proteins, p-Smad-2 and p-Smad-3, have been shown to interact with DROSHA and DICER to regulate the processing of miR-21 in cardiac fibroblasts [262, 263]. Mature miR-21 has been implicated in the development of RIF in several tissues [264–267]. In endothelial cells, H_2_O_2_ treatment downregulates the expression of DICER [268–270]. However, in hepatic stellate cells (HSC), inhibition of DICER suppresses HSC activation as well as ECM expression [271]. It is unknown if ROS are directly involved in PTMs of DROSHA and DICER activity. However, downregulation of DICER prevents the generation of ROS by lowering expression of the p47phox protein, which is a part of the NOX2 complex that generates ROS [272]. Therefore, there exists a close relationship between the miR-processing proteins, an active TGF-*β* signaling pathway, and ROS that needs to be further investigated in the context of RIF.

Following radiation, ten miR species have been found to be upregulated: let-7d, let-7g, let-7i, miR-26b, miR-663, let-7e, miR-15b, miR-21, miR-768-3p, and miR-768-5p. Seven miRs were found to be downregulated: miR-24, let-7a, miR-100, miR-125b, miR-222, let-7b, and miR-638 in normal human fibroblasts [250]. Out of these 17 miRs, changes in intracellular levels of hydrogen peroxide have been associated with altered expression of let-7d, let-7b, let-7e, miR-15b, miR-768-3p, miR-768-5p, miR-24, miR-21, and miR-638. Some miRs such as the miR-29 family members are not directly regulated by changes in ROS and are dependent on the TGF-*β* signaling pathway. MiR-29 family members are downregulated after radiation, which leads to an increase in expression of type I collagen genes that contribute to the development of RIF [273]. Further, loss of radioprotective miR-140 is observed in human lung fibroblasts, which is known to regulate the TGF-*β* signaling pathway and expression of fibronectin [274]. These miRs could potentially drive acute and chronic changes in molecular connections to combat oxidative stress during fibrosis [275].

Treatment with a thiol antioxidant, cysteine, prevents changes in the expression of some of the above miRs initiated by ionizing radiation [250]. The potential to regulate miR expression using locked nucleic acid- (LNA-) modified anti-miR inhibitors in combination with antioxidants is an attractive avenue for prevention of RIF [264]. Moreover, these miRs can be used as potential biomarkers for patients at risk of developing RIF [276–279].

Apart from miRs, other noncoding RNAs such as lncRNA, which are >200 nucleotides [280], and circRNA [281, 282] have also been shown to be dysregulated in RIF. lncRNAs play a role in epigenetic regulation by forming complexes with chromatin-modifying proteins. However, these RNA molecules have not been extensively studied in the context of changing oxidative stress. In normal human bronchial epithelial cells, overexpression of long intergenic radiation-responsive RNAs (LIRRs), noncoding RNAs, increased radiosensitivity through a DNA damage response (DDR) signaling mechanism that is p53 dependent [280]. Similarly, lnc-RI is a radiation-inducible lncRNA molecule involved in radiation-induced DDR [283]. In hepatic stellate cells, 179 circRNAs were found to be upregulated and 630 circRNAs were downregulated after irradiation [281]. Inhibition of hsa-circ-0071410 has been shown to attenuate radiation-induced hepatic stellate cell activation [281]. Two other circRNAs, KIRKOS-73 and KIRKOS-71, are upregulated following radiation exposure and can serve as a diagnostic radiotherapy biomarkers [282]. However, the role of these noncoding RNAs have not been studied in the context of ROS-mediated development of RIF. We do not know whether the use of antioxidants influences the expression of these molecules.

## 7. Conclusion

**Figure 2:**
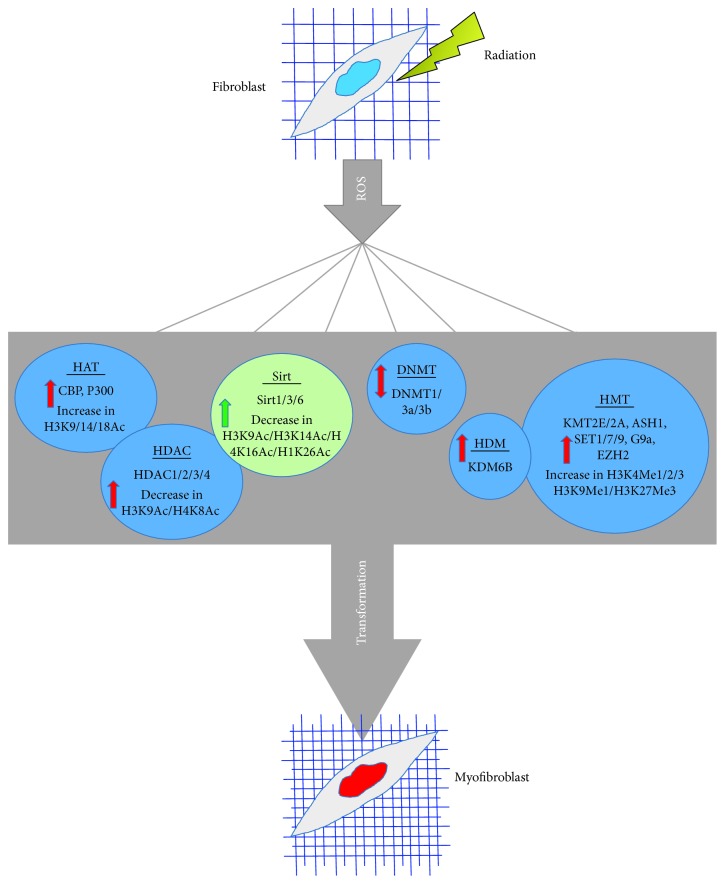
Changes in expression or activity of chromatin-modifying proteins that are redox sensitive, which lead to epigenetic reprogramming and transformation of fibroblast cells to myofibroblast cells after radiation. The red arrow indicates the increase or decrease in expression or activity driving transformation to myofibroblast. The green arrow indicates the increase in expression or activity preventing transformation to myofibroblast.

Radiation therapy leads to the development of RIF and decreases the overall quality of life of irradiated cancer patients. ROS is one of the main drivers of epigenetic reprogramming of myofibroblasts, and targeting ROS could prevent many of the changes associated with fibrosis, as shown in Figure 2. To treat and prevent RIF, there are several strategies that can be used including inhibition of epigenetic modulators, inhibition of the TGF-*β* signaling pathway [284, 285], or inhibition of ROS, using antioxidants as shown in Table 1. Targeting the TGF-*β* signaling pathway or targeting the epigenetic modifications directly can prevent the epigenetic reprogramming of fibroblast cells and RIF. However, the main problem with these strategies is that there are side effects due to lack of specificity. Globally reducing epigenetic factors or TGF-*β* signaling can result in damage to other cells or organs not affected by RIF. However, increasing the antioxidant capacity of cells to physiologically relevant levels during and after radiation therapy is an ideal strategy to prevent RIF with minimal side effects. As discussed above, antioxidants also prevent the activation of the TGF-*β* signaling pathway and/or epigenetic modifications observed after radiation exposure. Therefore, removing or scavenging ROS by natural antioxidant compounds and/or mimics of antioxidant enzymes that are safe and well tolerated for clinical use may have significant potential to prevent RIF safely in patients.

Several different types of antioxidants and antifibrotic agents have demonstrated efficacy in preventing radiation damage and inhibiting acute molecular changes that drive the fibrotic phenotype in a variety of RIF animal models (see Table 1). Recent studies using small molecule antioxidants that mimic SOD activity, MnTE-2-PyP or MnTnBuOE-2-PyP, protect from acute and chronic fibrosis by preventing fibroblast activation and underlying reprogramming into activated myofibroblasts [286, 287]. For this reason, MnTnBuOE-2-PyP is currently in clinical trials as a radioprotector for several kinds of cancer [288–290]. In addition, another SOD mimic, GC4419, has also been shown to be an effective radioprotector and is in clinical trials for head and neck cancers. Given that these molecules do not protect tumors from radiation damage, these SOD mimics are a very promising therapy for the prevention of RIF. We predict that in the near future, these compounds will be available for patients to protect from RIF and potentially treat other fibrotic disorders by mitigating the epigenetic changes that drive fibrosis.

## Figures and Tables

**Table 1:** Antioxidants/antifibrotic agents used to prevent radiation-induced damage and fibrosis.

Antioxidant/antifibrotic agents	Region	Radiation dose/animals	Dose	Effects	Reference
AEOL 10150 (catalytic SOD mimic)	Lung	28 Gy/rats	10-30 mg/kg/day, for 10 weeks	Inhibits TGF-*β* signaling	[291]

Alpha-lipoic acid	Small intestine	15 Gy/mice	100 mg/kg, 3 days before radiation	Reduces inflammation and cell death and reduces p-NF-*κ*B, MMP9, and MAPK signaling and facilitates regeneration of vitamins C and E and elevates glutathione levels [292]	[293]
	Thyroid	18 Gy/rats	100 mg/kg, 24 h before radiation	Inhibits TGF-*β* signaling	[115]
	Salivary gland	18 Gy/rats	100 mg/kg, 24 h before irradiation	Reduces oxidative stress by inhibiting gp91 mRNA expression	[294]

Amifostine (WR-2721)	Head and neck	20–70 Gy/humans	200 mg/m^2^ to 400 mg/m^2^	Thiol compound and free radical scavenger; reduces oxidative radicals and prevents xerostomia (dry mouth) postradiation.	[295, 296]
	Heart	22.5 Gy/rats	160 mg/kg, 15 minutes before radiation	Reduces cardiac damage	[297]
	Heart	18 Gy/mice	200 mg/kg, 30 minutes before radiation	Prevents vasculitis and vascular injury	[298]
	Kidney	15 Gy	200 mg/kg, 30 minutes before radiation	Prevents glomerular and tubular changes and interstitial fibrotic lesions postradiation	[299, 300]

Atorvastatin	Kidney	2 Gy/mice	50 mg/kg/day for 1 week	Reduces the levels of oxidative stress biomarkers	[301]

CpG oligodeoxynucleotide	Lung	15 Gy/mice	50 *μ*g CpG-ODN	Prevents radiation-induced pulmonary fibrosis by shifting the imbalance of Th1 and Th2 responses	[302]

Curcumin	Lung	18 Gy/rats	200 mg/kg/day, 1 week before radiation	Boosts antioxidant defenses by increasing HO-1, prevents COX-2 upregulation, and inhibits proinflammatory cytokines and NF-*κ*B signaling	[303]
	Lung	13.5 Gy/mice	1% or 5% (*w*/*w*)	Prevents radiation-induced pulmonary fibrosis and reduces LPS-induced TNF-*α* production	[304]

Erdosteine	Whole body/kidney	5 Gy/rats	100 mg/kg/day, 1 week before irradiation by gastric tube	Inhibits production of proinflammatory cytokines TNF-*α*, IL-1, IFN*γ*, and IL-6	[305]

Eukarion-189 (catalytic SOD catalase mimic)	Lung	10 to 20.5 Gy/rats	30 mg/kg, 30 minutes before radiation	Inhibits TGF-*β* signaling	[306]

Eukarion-207 (catalytic SOD catalase mimic)	Lung	12 Gy/rats	8 mg/kg/day	Reduces oxidative damage, TGF-*β*, and NF-*κ*B signaling and activated macrophages	[307]

Flaxseed	Lung	13.5 Gy/mice	10% (*w*/*w*)	Reduces expression of lung injury biomarkers (Bax, p21, and TGF-*β*) and contains omega-3 fatty acids and lignans with antioxidant properties	[308]

Follistatin	Hindlimb	35 Gy/mice	4 *μ*g, 24 hours before, 2 days after radiation, and then 3/week over 6 months	Inhibits TGF-*β* signaling	[285]

GC4401	Whole body/liver	2 × 2 Gy/mice	2 mg/kg before every fraction	Protects the liver in Sirt3^−/−^ animals from radiation-induced injury	[309]

GC4419	Oral cavity	60 to 72 Gy/humans	15 to 112 mg/day, 60 min before radiation for 3 to 7 weeks	Reduces the frequency and duration of oral mucositis	[310]

Genistein (isoflavone)	Lung	12 Gy/rats	50 mg/kg/day	Reduces oxidative damage, TGF-*β*, and NF-*κ*B signaling and activated macrophages and fibrosis	[307]

Ginger extract	Kidney	2, 4, and 8 Gy/rats	50 mg/kg/day for 10 days	Alleviates functional and structural alterations in the kidney due to antioxidant and anti-inflammatory effects	[311]

Gingko biloba	Whole body	8 Gy/rats	50 mg/kg/day, 15-day pretreatment	Attenuates irradiation-induced oxidative organ injury, by preventing an increase in LDH and TNF-alpha levels	[312]
	Eye	5 Gy/rats	40 mg/kg/day, 3 days pretreatment and up to 7 days postradiation	Prevents increase in xanthine oxidase (XO) activity postradiation	[313]
	Whole body	6 Gy/rats	50 and 100 mg/kg/day for 7 days	Corrects the metabolic disturbances induced in the brain by lowering dopamine, calcium, and zinc contents while increasing iron content and restores the activities of lactate dehydrogenase and cholinesterase enzymes	[314]

GTS-21 (*α*7-nAChR agonist)	Lung	12 Gy/mice	4 mg/kg/day	Reduces TNF-*α*, IL-1*β*, and IL-6 production in serum via inhibition of NF-*κ*B and downregulates TLR-4 and HMGB1 expression in the lungs and reduces ROS levels and HIF-1*α* expression along with inhibition of NOX1 and NOX2 expression	[315]

Hesperidin	Heart	18 Gy/rats	100 mg/kg/day for 7 days	Decreases inflammation, fibrosis, mast cell and macrophage numbers, and myocyte necrosis after radiation	[316]

JP4-039 (TEMPOL)	Skin/leg	35 Gy/mice	50 *μ*L of formulation, 0.5, 24, and 48 h after radiation	Reduces radiation-induced skin damage	[317]

KL4 surfactant (21-amino acid peptide)	Lung	13.5 Gy/mice	120 mg/kg twice daily	Reduces lung inflammation and oxidative stress	[318]

Matrine (alkaloid)	Whole body	6-7 Gy/rats	30, 10, and 3 mg/kg/day, 3 days before or after radiation	Reduces radiation-induced damage by altering 21 pathways	[319]

Melatonin	Lung	18 Gy/rats	100 mg/kg once 30 minutes before radiation	Reduces lipid peroxidation product malondialdehyde	[320]

MnTnHex-2-PyP (catalytic SOD mimic)	Lung	28 Gy/rats	0.05 mg/kg/day for 2 weeks, 2 h postradiation	Decreases HIF-1alpha, TGF-*β*, and VEGF A expression after radiation	[321]
	Lung	28 Gy/rhesus monkeys	0.05 mg/kg twice daily for 2 months	Prevents radiation injury in the lungs	[322]

MnTE-2-PyP Or AEOL 10113 (catalytic SOD mimic)	Prostate	10 Gy/mice	6 mg/kg/day, day 1 to 16	Inhibits TGF-*β* signaling and protects against decreases in RBC counts, hemoglobin, and hematocrit	[323]
	Pelvic region	20-30 Gy/rats	5 mg/kg/week, 1 h before radiation	Ameliorates both acute and chronic radiation proctitis	[324]
	Pelvic region	37.5 Gy/mice	10 mg/kg/week, 24 h before radiation; for the first two weeks, 3 times/week at a dose of 5 mg/kg	Reduces collagen deposition, inflammation, senescence, and fibroblast to myofibroblast differentiation and upregulates NQO1 expression	[286]
	Lung	28 Gy/rats	6 mg/kg/day, 15 min before radiation	Inhibits TGF-*β* signaling	[325]
	Lung	28 Gy/rats	6 mg/kg/day for 10 weeks	Decreases HIF-1alpha, TGF-*β*, and VEGF A expression after radiation	[326]

MnTnBuOE-2-PyP5 or BMX-001 (catalytic SOD mimic)	Brain	5 Gy/mice	1.5 mg/kg, twice daily, for 14 days	Protects hippocampal neurogenesis	[288]
	Brain	8 Gy/mice	1.6 mg/kg, twice daily, 24 h before radiation	Protects the brain from negative effects of cranial irradiation	[327, 328]
	Colon	2 Gy/mice	0.25 *μ*M every 3 days, for *in vitro* studies	Prevents activation and increase in cell size of fibroblast cells from the colon	[287]

N-Acetyl cysteine (NAC)	Whole body	18 Gy/mice	500 mg/kg/day, 3 days before and up to 3 days postradiation	Protects the lung and red blood cells from glutathione depletion following irradiation	[329]
	Whole body	6 Gy/rats	1000 mg/kg, 15 min before radiation	Protects rat femoral bone marrow cells from radiation-induced genotoxicity and cytotoxicity	[330]
	Abdomen	10 Gy/rats	300 mg/kg/day	Alleviates the negative effects of radiotherapy on incisional wound healing by means of reducing oxidative stress markers	[331]
	Abdomen	20 Gy/mice	300 mg/kg/day, for 7 days	Prevents gastrointestinal injury, damage to bone marrow stromal cells, and radiation-induced acute death	[326]

Plasminogen activator inhibitor-1 (PAI-1) truncated	Lung	30 Gy/mice	5.4 *μ*g/kg/day for 18 weeks beginning 2 days before radiation	Prevents RIF with increased fibrin metabolism, enhanced matrix metalloproteinase-3 expression, and reduced senescence in type 2 pneumocytes	[332]

Pirfenidone	Lung	16 Gy/mice	300 mg/kg/day for four weeks	Inhibits TGF-*β* signaling	[333]
	Intestine	20 Gy/mice	200 and 400 mg/kg/day for 12 weeks		[334]
	Head and neck	60-72 Gy/humans	800 mg three times/day	—	[335]

Podophyllotoxin and rutin combination (G-003M)	Lung	11 Gy/mice	5 mg/kg once	Reduces radiation-induced oxidative and inflammatory stress	[336]

Polydatin	Lung	15 Gy/mice	100 mg/kg/day	Exerts anti-inflammation and antioxidative properties through Nrf2 signaling and Sirt3 upregulation	[213]

Quercetin	Intestine	13 Gy/mice	100 mg/kg/day for 6 days before and after radiation	Inhibits TGF-*β* signaling	[337]
	Skin/hind leg	35 Gy and 10 Gy/mice	Quercetin-formulated chow (1% by weight)		[338]

Resveratrol	Intestine	7 Gy/mice	40 mg/kg/day, 1-day pretreatment and up to day 5	Prevents intestine damage via the activation of Sirt1, improves intestinal morphology, decreases apoptosis of crypt cells, maintained cell regeneration, ameliorated SOD2 expression and activity, regulates Sirt1, and acetylated p53 expression that is perturbed by irradiation	[339]
	Whole body	3 Gy/mice	100 mg/kg/day, 2 days pretreatment and up to 30 days	Reduces radiation-induced chromosome aberration frequencies	[340]
	Salivary gland	15 Gy/mice	20 mg/kg/day	Inhibits TGF-*β* signaling and protects the salivary glands against the negative effects of irradiation	[341]
	Ovary	21 Gy/rats	25 mg/kg/day for 2 weeks	Counteracts the effect of radiation and upregulates the gene expression of PPAR-*γ* and Sirt1, leading to inhibition of NF-*κ*B-provoked inflammatory cytokines	[191, 342]
	Whole body/hematopoietic stem cell	6 Gy/mice	20 mg/kg/day for 7 days before and then up to 30 days postradiation	Protects from radiation-induced injury, in part, via activation of Sirt1	[343]
	Skin	35 Gy/mice	1% by weight	Inhibits TGF-*β* signaling	[338]
	Lung	13 Gy/mice	100 mg/kg/day for 7 days	Prevents lung injury by reducing inflammation and fibrosis	[344]

rhNRG-1*β*	Heart	20 Gy/rats	15 *μ*g/kg, 3 days before and 7 days after radiation	Prevents fibrosis and preserves cardiac function via the ErbB2-ERK-Sirt1 signaling pathway	[345]

Silibinin	Breast	46.8-50.4 Gy/humans	400 IU for 6 months	Vitamin E may be clinically useful in preventing fibrosis after radiation in high-risk patients	[346]

SOD gliadin	Hind leg/skin	25 Gy/mice	10000 units/kg/day for 8 days	Reduces dermal thickness and fibrosis after irradiation	[347]

Soy isoflavones	Prostate	73.8 to 77.5 Gy/humans	200 mg tablet containing 50 mg soy isoflavones (genistein, daidzein, and glycitein at a ratio of 1.1 : 1 : 0.2)	Reduces the urinary, intestinal, and sexual adverse effects in patients with prostate cancer receiving radiation therapy	[348]
	Lung	12 Gy/mice	50 mg/kg/day, 3 days before and up to 4 months after radiation	Mitigates inflammatory infiltrates and radiation-induced lung injury	[349]
	Lung	10 Gy	250 mg/kg/day, 3-day pretreatment	Inhibits the infiltration and activation of macrophages and neutrophils induced by radiation in the lungs	[350]
	Lung	12 Gy/mice	250 mg/kg/day, 3-day pretreatment and up to 4 months after radiation	Inhibits the infiltration and activation of macrophages and neutrophils induced by radiation in the lungs	[349]

Taurine	Lung	14 Gy/mice	32 mg/kg/day	Inhibits TGF-*β* signaling; taurine essential amino acid is involved in osmoregulation, antioxidation, detoxification, membrane stabilization, neuromodulation, cardiac function, and central nervous system development	[351]
	Brain	6 Gy/rats	2 oral doses of 500 mg/kg/day for 2 weeks	Taurine has antioxidant, anti-inflammatory, and antiapoptotic effects	[352]
	Sperm cells	8 Gy/mice spermatocytes (GC-2 cells)	40 mM	Activates Nrf2/HO-1 signaling	[353]

Vitamin E	Lung & heart	20 Gy/rats	2.5% of diet 2 weeks before radiation or 150 mg injected 4 h before radiation	Protects lungs and heart tissues from radiation damage	[354]
	L4ung	14 Gy/rats	1.1 mg/day dissolved in 0.1 mL olive oil injected	Protects against the development of RIF	[355]
	Whole body	9.2 Gy/mice	50 mg/kg 24 h before radiation	Protects against acute radiation syndrome	[356]

SKI2162	Hind limb	22 Gy/mice	10 mg/kg/day, 5 times/week	An inhibitor of the TGF-*β* type I receptor (ALK5) and inhibits radiation-induced fibrosis	[357]

GV1001 (hTERT peptide fragment)	Skin	6 Gy/mice	1 mg/kg/day and 5 mg/kg/day for 4 weeks	Suppresses TGF-*β* signaling	[358]

XH-103	Intestine	11 Gy/mice	200 mg/kg, 1 before radiation	Prevents damage to the intestinal crypt-villus structure	[359]
